# Robust crowd anomaly detection via hybrid ensemble learning for real-world surveillance

**DOI:** 10.1038/s41598-025-27408-9

**Published:** 2025-11-24

**Authors:** Doaa Mabrouk, Manal A. Abdel-Fattah, Ahmed Taha

**Affiliations:** 1 Software Engineering Department, Faculty of Engineering & Technology, Egyptian Chinese University, Cairo, Egypt; 2 Information Systems Department, Faculty of Computers and Artificial Intelligence, Helwan University, Helwan, Egypt; 3 Computer Science Department, Faculty of Computers and Artificial Intelligence, Benha University, Benha, Egypt

**Keywords:** Crowd anomaly detection, YOLOv7, Random forest classifier, Gradient boosting classifier, Adam optimizer, Video surveillance cameras, Machine learning, Computer science, Information technology, Software

## Abstract

Crowd Anomaly Detection (CAD) is a crucial task in intelligent surveillance systems, designed to enhance public safety in densely populated environments. While existing approaches, including deep learning and traditional machine learning models, have shown promise, they often suffer from limited generalizability, high computational cost, or poor performance on small-scale datasets. To address these gaps, this study proposes a novel hybrid ensemble learning framework that integrates the speed and accuracy of YOLOv7 for real-time crowd detection with the robustness and interpretability of classical ensemble classifiers, namely Random Forests (RFs) and Gradient Boosting (GB). The model extracts spatial and motion features via optical flow from YOLO-detected regions, reduces dimensionality, and classifies behaviors as normal or abnormal. The ensemble is optimized using the Adam optimizer to improve performance on data-constrained surveillance scenarios. Experimental results on the University of Minnesota “UMN” benchmark dataset show a near-perfect accuracy of 99.89%, while evaluation on a custom real-world supermarket dataset demonstrates strong generalizability and robustness. These results establish a new standard for small-scale CAD and demonstrate the practical applicability of the proposed framework for intelligent, real-time surveillance.

## Introduction

**Fig. 1:**
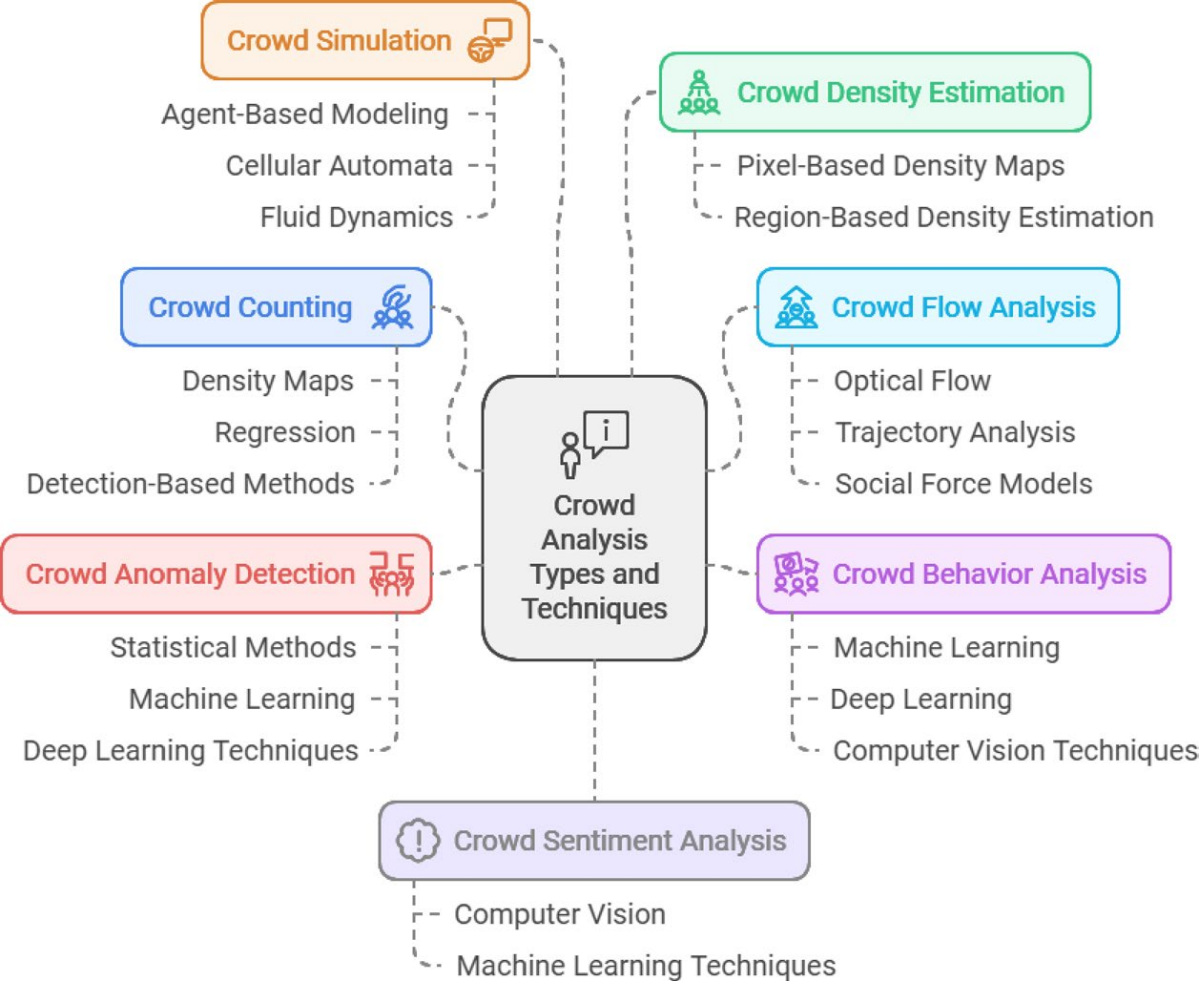
Crowd analysis types and techniques.

Crowd Analysis (CA) and detection have emerged as two of the most crucial research domains in Computer Vision (CV) and Machine Learning (ML). Figure 1 shows the core CA Types and Techniques which organizes in six different areas: Crowd Simulation, which encompasses agent-based modeling, cellular automata, and fluid dynamics; Crowd Density Estimation, achieved with pixel-based density maps and region-based estimation; Crowd Flow Analysis, employs optical flow, trajectory analysis, and social force models; Crowd Behavior Analysis, often employing machine learning, deep learning, and computer vision techniques; Crowd Anomaly Detection, approached with statistical methods, machine learning, and deep learning; and finally, Crowd Counting, which leverages density maps, regression, and detection-based methods. These diversities are essential in a broad spectrum of applications, including public safety, urban planning, marketing, surveillance, and event management. CA^1^, on the other hand, has been highly relevant in several areas. For public safety, it aids in identifying anomalies and predicting crowd behaviors, which helps prevent disasters, accidents, and security breaches. In urban planning, it aids in planning the city based on crowd dynamics and infrastructure design. For marketing purposes, crowd analysis offers valuable insights into consumer trends and preferences. Surveillance serves as a method of observing public spaces to recognize potential dangers and reduce the workload for human operators. In event management, understanding crowd dynamics helps optimize event planning and resource allocation.

CAD has recently become one of the most critical topics due to the growing demand for robust and effective surveillance systems in the context of public safety. Crowds, whether in public areas, transportation centers, or at massive events, are complex and dynamic situations with potential dangers^2^. CAD has become a crucial area of research, with applications spanning public safety, urban management, healthcare, and security. It can identify unusual behaviors, such as sudden crowd dispersal, stampedes, unauthorized intrusions, or medical emergencies. In smart cities, it aids in optimizing pedestrian flow, preventing overcrowding in transportation hubs, and enhancing event management. Security applications include real-time surveillance for crime detection, terrorism prevention, and border monitoring. Additionally, it plays a vital role in epidemic control by monitoring social distancing and predicting disease transmission patterns. In malls, anomaly detection can help prevent shoplifting and optimize customer flow, leading to improved store layouts. Urban planning is enhanced by an understanding of crowd behavior, which aids in the design of infrastructure and the effective management of traffic flow. Event management utilizes these technologies to ensure safety and optimize resource utilization in managing large-scale events. Additionally, in security-sensitive environments, such as airports and critical infrastructure, anomaly detection in crowds enhances surveillance measures, enabling an early response to security breaches.

CAD faces several challenges, including the diversity of anomalies, which can manifest in various forms, such as isolated individuals, group formations, or unusual crowd movements. Occlusions pose another difficulty, as people in crowds often block each other from view, making it complex to track individual behaviors. Camera variations also present challenges; factors such as camera angles, resolution, and lighting conditions can alter the appearance of crowded situations. Finally, real-time processing is essential, as anomaly detection in real-time video streams demands highly efficient algorithms.

Several research efforts have been dedicated to addressing these challenges. Traditional methods, such as background subtraction, optical flow, and statistical approaches^3,4^, have been widely explored. Additionally, Machine Learning (ML) techniques, including Support Vector Machines (SVM)^5^, Hidden Markov Models (HMM)^6,7^, and Neural Networks, have been applied to anomaly detection. However, these methods often struggle with robustness and generalization in real-world scenarios. Deep learning-based approaches, such as Convolutional Neural Networks (CNN)^8^, Recurrent Neural Networks (RNN), and Generative Adversarial Networks (GAN)^9^ have shown promise but typically require large, labeled datasets, which are often scarce for rare anomalies in surveillance environments.

To address these limitations, our research focuses on developing a more efficient and reliable crowd anomaly detection system to enhance public safety and security. We introduce a novel hybrid approach that combines the strengths of RFs and GB classifiers. RFs offer robustness against noise, interpretability, and effectiveness in handling high-dimensional data, while GB excels in prediction accuracy and capturing complex patterns. By integrating these complementary techniques, our method achieves a balance between performance and interpretability, ensuring high accuracy and efficiency in real-world surveillance applications. Furthermore, we optimize our approach using the Adam optimizer, which enhances performance on small-scale datasets—an essential factor in real-world anomaly detection scenarios.

The contributions of this work are summarized as follows:

- Development of a Hybrid Anomaly Detection Method: The paper introduces a novel hybrid model that combines Random Forests (RFs) and Gradient Boosting (GB) classifiers. This integration leverages the interpretability and robustness of RFs, combined with the high predictive power of GB, to offer a balanced solution for anomaly detection in crowded scenes.
- Optimization with the Adam Optimizer: The paper presents a fine-tuned method using the Adam optimizer, which is explicitly tailored for detecting anomalous behaviors in small-scale crowd datasets, thereby improving generalization and accuracy. This optimization enhances the model’s ability to handle the variability and noise commonly found in surveillance settings.
- State-of-the-Art Performance on Benchmark Dataset: The proposed method achieves a near-perfect accuracy of 99.89% on the UMN scenes, with consistently high accuracy across all scenes. This result surpasses existing methods, particularly for small-scale crowd datasets, establishing a new benchmark in anomaly detection performance.
- Evaluation on a Real-World Custom Dataset: The proposed method is rigorously tested on a self-collected and annotated dataset of surveillance videos from supermarket environments. This dataset, specifically curated to include diverse normal and abnormal activities, ensures a robust evaluation of the model’s practical applicability in real-world scenarios. The results demonstrate the method’s effectiveness in detecting anomalies in realistic, noisy, and dynamic settings, further validating its adaptability and generalization capability.

The remainder of this paper is organized as follows. “Related Work” reviews existing approaches and relevant studies in the field. “Proposed Method” describes the hybrid framework that integrates Random Forests, Gradient Boosting classifiers, and the Adam optimizer. “UMN Dataset” introduces the benchmark dataset used for initial evaluation, while Custom Dataset presents the supermarket surveillance data specifically collected for this research. “Results and Discussion” provides the experimental setup, performance analysis, and comparative results. Finally, “Conclusion and Future Work” summarizes the key findings and outlines potential directions for future research.

## Related work

Abnormal crowd behavior detection has become a critical research area, aiming to enhance public safety through the use of advanced surveillance techniques. Various methodologies, developed by integrating deep learning and multi-source information fusion, have improved detection accuracy and efficiency. The detection techniques, such as the YOLO network, achieve high accuracy, reaching up to 0.99 for object detection in heavy crowds. However, it takes more processing time compared to YOLOv3 tiny, which is 0.75 in^10^. Multi-Source Information Fusion proposes a new technique that fuses depth-wise separable convolutional neural networks (DWS-CNNs) and LiteFlowNet with Multi-Object Tracking by Segmentation (MOSSE) tracking. It improves the precision of detection by incorporating fuzzy logic and weighted averages in^11^. CNN and Bidirectional Long Short-Term Memory (Bi-LSTM), as presented in^12,13^, proposed a model that could classify normal and abnormal behaviors with an accuracy of 98.5%. The model used a custom dataset explicitly designed for real-time surveillance applications. These challenges pertained to video quality and occlusions. Other techniques include transferring pre-trained Visual Geometry Group (VGG) filters and utilizing Support Vector Machines (SVMs) to address challenges such as poor image quality and occlusions, achieving a recognition accuracy of 94.3%^14^. Although these methods have enhanced crowd anomaly detection, they often face trade-offs between accuracy, computational efficiency, and robustness in real-world scenarios. Key challenges, including high computational costs, sensitivity to video quality, and limitations in capturing detailed temporal dynamics, persist as critical research areas. Future advancements should prioritize developing approaches that effectively balance these factors, ensuring both efficiency and reliability in complex surveillance environments.

CAD using ML has emerged as a crucial research domain, particularly for enhancing public safety in densely populated environments. Various machine learning-based approaches have been proposed to detect and classify anomalous behavior by leveraging video image processing techniques. These methods typically involve extracting behavioral features from video data, such as movement patterns (e.g., walking, falling), to train models for accurate classification. Background subtraction techniques are employed to eliminate static elements within video frames, thereby isolating dynamic behaviors and improving detection efficiency. Predictive neural networks have also been utilized to identify anomalies by comparing estimated and actual frames, achieving an accuracy of 97.7% on specific datasets. Performance metrics such as F1 score, recall, precision, and accuracy are commonly reported to assess the robustness of these models^15^. Additionally, CNNs have demonstrated effectiveness in image recognition tasks, offering enhanced detection accuracy with lower computational overhead compared to traditional models^16^.

While ML has significantly improved crowd anomaly detection, challenges such as occlusions in video frames and real-time processing remain critical issues that must be addressed. Research must focus on developing robust feature extraction techniques, training models on diverse datasets, and designing algorithms that can handle real-world complexities, including variations in lighting conditions, weather, and camera angles. Addressing these challenges is essential for enhancing the reliability and efficiency of CAD systems in dynamic surveillance environments.

Hybrid classifiers will play a significant role in detecting, tracking, and recognizing abnormal behaviors in dense crowds. Integrating various machine-learning-based approaches leads to higher accuracy and improved efficiency for any system. Recent works in this domain utilize hybrid classifiers comprising CNNs and RFs. The hybrid model, which integrates CNNs, specifically Residual Network-50 (ResNet-50), with RF, has proven very successful in detecting abnormal behaviors in both small and large crowds. This model achieved an average area under the curve (AUC) of 76.08% on the HAJJv2 dataset, surpassing the existing methods^17^. In^18^, Anomaly Detection and Localization: Combining methods using Adaptive Gaussian Mixture Model (AGMM) and You Only Look At Coefficients (YOLACT) has improved foreground detection, while YOLOV5 and DeepSORT effectively track the detected anomalies. While hybrid classifiers show promising results, occlusions and variations in crowd dynamics pose challenges as they rely on accurate detection. Such techniques require further refinement to be more reliable under varying environmental conditions. In^19^, a hybrid CNN-Transformer model is presented to improve feature extraction for anomaly detection in small-scale surveillance settings, highlighting the growing trend toward integrating attention mechanisms into classical vision pipelines.

A hybrid deep learning architecture has significantly improved anomaly event detection in surveillance videos by integrating spatial and temporal features. Recent studies have shown that two-stream models can achieve more accurate anomaly detection and classification performance. Two-stream architecture consists of spatial and temporal streams. These architectures primarily comprise spatial streams for extracting appearance features and temporal streams for capturing motion patterns. For example, consider the spatial feature extraction of models like YOLO-V4 and Visual Geometry Group-16 (VGG-16), with the Optical FlowNet used for extracting temporal features in^20,21^. Feature Fusion: The feature fusion among streams plays a crucial role in improving the detection performance. Late fuzzy fusion and joint reconstruction error are some methods involved in effectively merging these features in^21,22^. Recent studies such as^23,24^ also demonstrate the effectiveness of combining CNNs and LSTMs for spatiotemporal anomaly detection, achieving strong performance in dynamic surveillance environments.

Real-time crowd detection is one of the most critical research areas for which high-powered technologies, including drones and Internet of Things (IoT) devices, along with deep learning models^25^, are leveraged to monitor crowds effectively. In^26,27^, recent related studies emphasize various methodologies that help to raise detection accuracy and combat challenges posed by dynamic environments and variable crowd densities.

In^28^, the Airborne Crowd Anomaly Detection (Air-CAD) system is presented as a network of drones that perform crowd anomaly detection. The dynamic adjustment of drone positioning-based detection tasks enhances performance to 95.33% area under the receiver operating characteristic curve (AUROC) and reduces real-time inference latency to 0.47 s. The approach focuses on air-ground collaboration, where the processing can be done efficiently through edge devices. IoT sensors integrated with the YOLO model are showing much promise for real-time counting, while YOLO V8 neural architecture search (NAS) reported a state-of-the-art mean Average Precision (mAP) of 95.1% in^29^. The architecture of YOLO enables it to quickly detect and locate people, thereby facilitating effective crowd management and control. A novel method that utilizes adaptive thresholding and the Visual Background extractor (ViBe) to segment groups in medium-density crowds demonstrates improved efficiency in complex environments. While such achievements demonstrate strong potential for crowd analysis, real-time is also an area with many challenges that require further research and development of methods for highly dense scenarios. Real-time anomaly detection using YOLO variants has also been explored in^30^, which focuses on lightweight and efficient detection architectures suitable for embedded surveillance systems.

Table 1 summarizes the studies that consider crowd anomaly detection in video surveillance systems. Existing approaches can broadly be categorized into three classes: deep learning-based methods, traditional machine learning methods, and hybrid models. Although these methods have achieved reasonable success, several research gaps persist: Many deep learning methods lack interpretability and perform poorly on smaller datasets. Few models effectively combine real-time detection with robust classification under varying scene conditions. Most existing works overlook the potential of integrating classical ensemble techniques (e.g., RF, GB) with modern object detection frameworks^31^, such as YOLO, for CAD. In contrast to prior studies, our work introduces a hybrid ensemble anomaly detection framework that uniquely combines YOLOv7 for efficient and accurate crowd region detection with Random Forest and Gradient Boosting classifiers for behavior classification. This integration enables a scalable, interpretable, and high-performance system suitable for both benchmark and real-world surveillance scenarios. Unlike purely deep or purely shallow methods, our approach strikes a balance between computational efficiency, accuracy, and generalizability, especially on small-scale datasets, as demonstrated through extensive experiments on both the UMN dataset and a custom supermarket surveillance dataset.

**Table 1:** Summary of crowd anomaly detection using surveillance systems.

Ref #	Video analysis purpose	Applied techniques	Offline/real-time	Evaluation criteria	Result	Limitation
^9^	Comparison between two techniques for detection	YOLOv3 and YOLOv3 tiny	offline	Precision, accuracy	The two techniques showed comparable precision, with YOLOv3 achieving 0.99 and YOLOv3 tiny achieving 0.75.	Occlusion handling in dense crowds
^10^	Detecting abnormal crowd behavior in surveillance videos	Depth-wise Separable Convolutional Neural Network (DWS-CNN); a Combination of LiteFlowNet, MOSSE tracker, and DSC-GRU network.	offline	Precision, Accuracy	Enhanced accuracy and improved reliability	Challenges in generalizing to diverse crowd scenarios, computational complexity of fusing multiple information sources
^11^	Detecting abnormal behavior in crowded scenes	The transfer of the Deep filter bank of pre-trained VGG on ImageNet	offline	Accuracy	94.3% recognition accuracy	Handling diverse crowd behaviors and varying environmental conditions, generalizing to different datasets or real-world situations if the training data is not sufficiently diverse
^12^	Increase the efficiency of the detection	Background subtractor to extract the spatiotemporal features	offline	F1 score, Recall, Precision, and Accuracy	97.7% on a sample of the UCSD dataset (50 training and 48 test samples)	Generalizability may be limited, and the model’s interpretability raises concerns, making it difficult to understand the reasons behind anomaly detections.
^14^	Detecting, tracking, and recognizing spatiotemporal abnormal behaviors in both small and large-scale crowds	Hybrid classifiers combining convolutional neural networks (CNNs) and random forests (RFs)	offline	Accuracy	99.76% and 93.71% of the average area under the curves (AUCs) on UMN and UCSD, respectively	data acquisition and processing in the large-scale, often chaotic, Hajj setting
^16^	A two-stream hybrid deep learning architecture for anomaly event detection	YOLO-V4 and Visual Geometry Group-16 (VGG-16) with temporal features from FlowNe	offline	Accuracy	95.6% on the UCF crime dataset	Challenges include hyperparameter tuning and model optimization. The interpretability of a hybrid deep learning architecture could also pose a challenge for understanding the reasons behind specific anomaly detections.
^20^	Air-CAD enables real-time crowd anomaly detection by utilizing an edge-assisted multi-drone network.	Dynamic inference adjustment for person detection models. Edge devices offload feature analysis tasks from drones.	Real-time	Accuracy	Air-CAD achieves 95.33% AUROC for crowd anomaly detection. Real-time inference latency is within 0.47 s.	Network failures or latency issues, data security and privacy, and the computational limitations of edge devices
^21^	Integrated crowd counting system utilizing IoT sensors and the YOLO model for real-time crowd detection.	Fusion of IoT devices and YOLO object detection model. Analysis of YOLO V5, V8, and V8 NAS variants.	Real-time	Precision	YOLO V8 NAS achieves a mean average precision of 95.1%.	A lack of detailed analysis of the system’s performance in diverse environmental conditions and with varying behaviors; accuracy in counting individuals may be affected by occlusions and variations in crowd density. YOLO’s performance in dense crowds requires further analysis
^22^	Detecting and classifying abnormal crowd behavior in real-time video surveillance	CNN and BiLSTM	offline	Accuracy	98.5% for the custom dataset	Handling long-range dependencies in complex crowd behaviors with limited interpretability makes it challenging to understand the reasons behind specific anomaly detections.
^23^	Detect crowd densities, which can significantly assist in urban planning for smart cities	DCNNCDM-IUP	Real-time	Accuracy	accuracy value of 98.40% compared to existing DL models.	The interpretability of CNN-based models may be limited, as their reliance on visual data can be affected by environmental factors such as lighting and weather conditions, and the system’s privacy implications require careful consideration.

## Proposed method

**Fig. 2:**
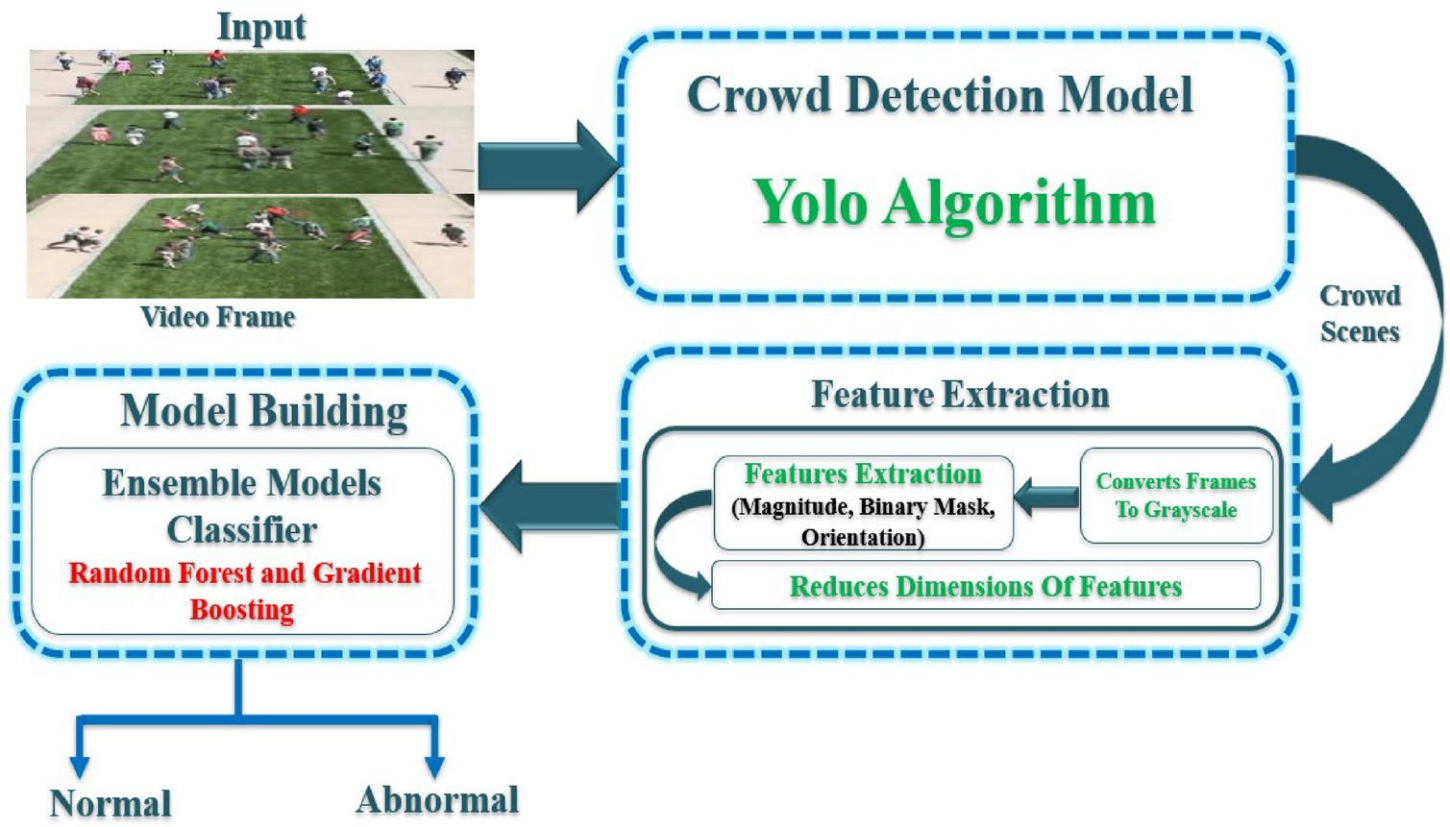
The block diagram of the proposed hybrid method.

This section presents the proposed hybrid method for detecting abnormal behavior, or “anomalies,” in crowded scenes. The hybrid ensembles learning framework for crowd anomaly detection processes video input through frame preprocessing. YOLOv7 performs crowd detection, followed by the extraction of both motion and spatial features from detected regions. These features are then concatenated and reduced in dimensionality before being fed into a hybrid ensemble of RFs and GB classifiers to classify behavior as either normal or abnormal. System architecture integrates deep learning with classical machine learning techniques—specifically, ensemble classifiers—to enhance the robustness of anomaly detection. As illustrated in Fig. 2, the proposed hybrid framework comprises three key stages: crowd detection, feature extraction, and model construction.

### Crowd anomaly detection

To identify individuals and crowded regions within a given video frame, we employ the YOLOv7 algorithm as the backbone of our crowd detection model. YOLOv7 is a real-time object detection technique renowned for its high speed and accuracy, making it well-suited for efficiently processing video frames. The output of this stage consists of detected crowd scenes, which are then passed to the feature extraction module for further processing. YOLOv7 can effectively detect regions of interest (ROIs) containing crowds. This step prevents the processing of irrelevant regions in the video frames later, thereby reducing computational overhead. The input consists of video frames captured from surveillance cameras or other sources, while the output includes detected crowd regions along with confidence scores indicating the likelihood of a crowd’s presence.

The YOLOv7 algorithm was selected for detecting crowded regions due to its superior speed, accuracy, and efficiency compared to traditional object detection methods. Unlike region-based approaches such as regions with convolutional neural networks (R-CNN) and Faster R-CNN, which require multiple passes over an image, YOLOv7 processes an entire frame in a single pass, significantly reducing computation time and enabling real-time detection. This makes it highly suitable for surveillance applications where timely anomaly detection is crucial. Additionally, YOLOv7 strikes a balance between precision and recall, minimizing both false positives and false negatives when identifying dense crowds. Its ability to detect multiple objects within a frame while maintaining high detection accuracy ensures reliable crowd identification, even under complex scenarios with varying lighting, occlusions, and diverse crowd formations. These advantages make YOLOv7 a robust choice for detecting crowded regions, enhancing the efficiency and effectiveness of the anomaly detection pipeline.

### Feature extraction

Once the crowd regions are identified, relevant features are extracted to capture movement dynamics and spatial characteristics. The feature extraction process involves four key steps:

- **Frame conversion to grayscale**: To reduce computational complexity and focus on essential structural information, all detected crowd frames are converted to grayscale.
- **Feature extraction**: Key features, including magnitude, binary masks, and orientation, are extracted to capture motion patterns and behavioral characteristics. The magnitude represents the intensity of motion within the crowd. Let $\:I(x,y,t)$ denote the grayscale intensity of a pixel at a spatial location $\:(x,y)\:$and time t. The optical flow equation assumes that the brightness of a moving object remains constant over time:

1 $$\:{I}_{x}u+{I}_{y}v+{I}_{t}=0$$

Where:

$\:{I}_{x}=\frac{\partial\:I}{\partial\:x}$. Spatial gradient along the x-axis.

$\:{I}_{y}=\frac{\partial\:I}{\partial\:y}$. Spatial gradient along the y-axis.

$\:{I}_{t}=\frac{\partial\:I}{\partial\:t}$. Temporal gradient (change in intensity over time).

$\:u$ and $\:v$: Components of the velocity vector along the x- and y-axes, respectively.

The magnitude of motion M(x, y) is then calculated as the Euclidean norm of the velocity vector:

2 $$\:M\left(x,y\right)=\sqrt{{u}^{2}+{v}^{2}}$$

The orientation captures the direction of motion within the crowd. The angle $\:\theta\:(x,y)$ of the motion vector with respect to the horizontal axis is given by:

3 $$\:\theta\:\left(x,y\right)=\text{arctan}\left(\frac{v}{u}\right)$$

Where:

- arctan: The arctangent function, which computes the angle in radians.
- $\:u$ and $\:v$: Components of the velocity vector along the x- and y-axes, respectively.

To ensure the orientation lies within the range $\:\left[\text{0,2}\pi\:\right]$, the following adjustments are made:

- If $\:u>0$ and $\:v>0$, $\:\theta\:(x,y)$ remains unchanged.
- If $\:u<0$, Add $\:\pi\:$ to $\:\theta\:(x,y).$.
- If $\:u>0$ and $\:v<0$, Add $\:2\pi\:\:$to $\:\theta\:(x,y).$.

These features represent motion, shape, and texture. It captures discriminative characteristics that differentiate between normal and abnormal crowd activities, serving as input for our classification models. Crucially, the extraction of these localized features, particularly the binary mask, implicitly performs in a form of crowd or individual localization by segmenting moving objects within the frame. The subsequent analysis focuses on these localized regions, allowing the model to analyze the behavior of specific parts of the crowd.

- **Dimensionality reduction**: Extracted features undergo dimensionality reduction to improve computational efficiency while retaining critical information relevant to anomaly detection. The feature space is projected down into a lower-dimensional space.
- **Temporal feature extraction**: This process extracts information on motion and temporal patterns throughout the entire video sequence. The process can be accomplished using a variety of techniques, each employing optical flow or temporal difference. These temporal features provide crucial context for classifying the observed crowd behavior.

Normal behaviors are typically characterized by smooth, consistent motion patterns, such as walking in a defined direction, standing still, or browsing. These behaviors result in lower motion magnitude, regular orientation flow, and stable binary masks in the optical flow maps. Abnormal behaviors, on the other hand, are identified by sudden changes in velocity or direction, such as running, loitering, abrupt stops, chaotic movement, or unauthorized entry. These are reflected in high motion magnitudes, irregular or rapidly changing orientations, and fragmented or erratic binary masks. These motion and spatial features are extracted using optical flow-based analysis and are further reduced in dimensionality before classification. We emphasize that these features were selected specifically to highlight behavioral deviations that signal anomalies in crowded surveillance footage.

### Model building

The final stage involves training an ensemble-based classification model to distinguish between normal and abnormal crowd behavior. We leverage a combination of RFs and GB classifiers to improve detection accuracy and model interpretability. RFs offer robustness against noise, effectively handle high-dimensional data, and provide interpretability, making them suitable for real-world surveillance applications. On the other hand, GB enhances predictive accuracy by sequentially improving weak classifiers and capturing complex behavioral patterns in the crowd. By integrating these complementary classifiers, our hybrid model achieves a balance between performance, efficiency, and reliability. The final classification output categorizes detected crowd behavior as either normal or abnormal, facilitating real-time surveillance and proactive security measures. This hybrid approach enhances anomaly detection by leveraging deep learning for accurate crowd detection while utilizing ensemble machine learning techniques for effective classification, ultimately improving safety and surveillance capabilities in crowded environments.

- **Model building and training**:
- **Data scaling**:
  - Normalization: Values are scaled within a specific range, from 0 to 1, which improves convergence for the model.
- **Data splitting**:
  - Train-Test Split: The dataset is divided into training and testing sets here. Later, the training set is used to train the model, and the testing set is used for evaluation.
  - Cross-Validation: A resampling approach that enhances the robustness of model evaluation by dividing the data into several folds, training various subsets, and averaging their performance.
- **Ensemble models**:
  - Random Forest: Train the Random Forest classifier using the preprocessed data. Random Forest constructs a large number of decision trees, each trained on a random subset of the data. The final prediction is made by combining the predictions of all trees via voting. Random Forest is robust to noise and overfitting, making it suitable for handling complex crowd data.
  - Gradient Boosting: Train a classifier using Gradient Boosting on the same data. GB trains models sequentially, where each successive model tries to correct the mistakes of the previous ones.
  - Hybrid method: the predictions are combined using weighted voting or other methods such as stacking on RFs and GB. Use Adam as the optimizer for both models.
- **Hybrid ensemble classifier structure**.

**Fig. 3:**
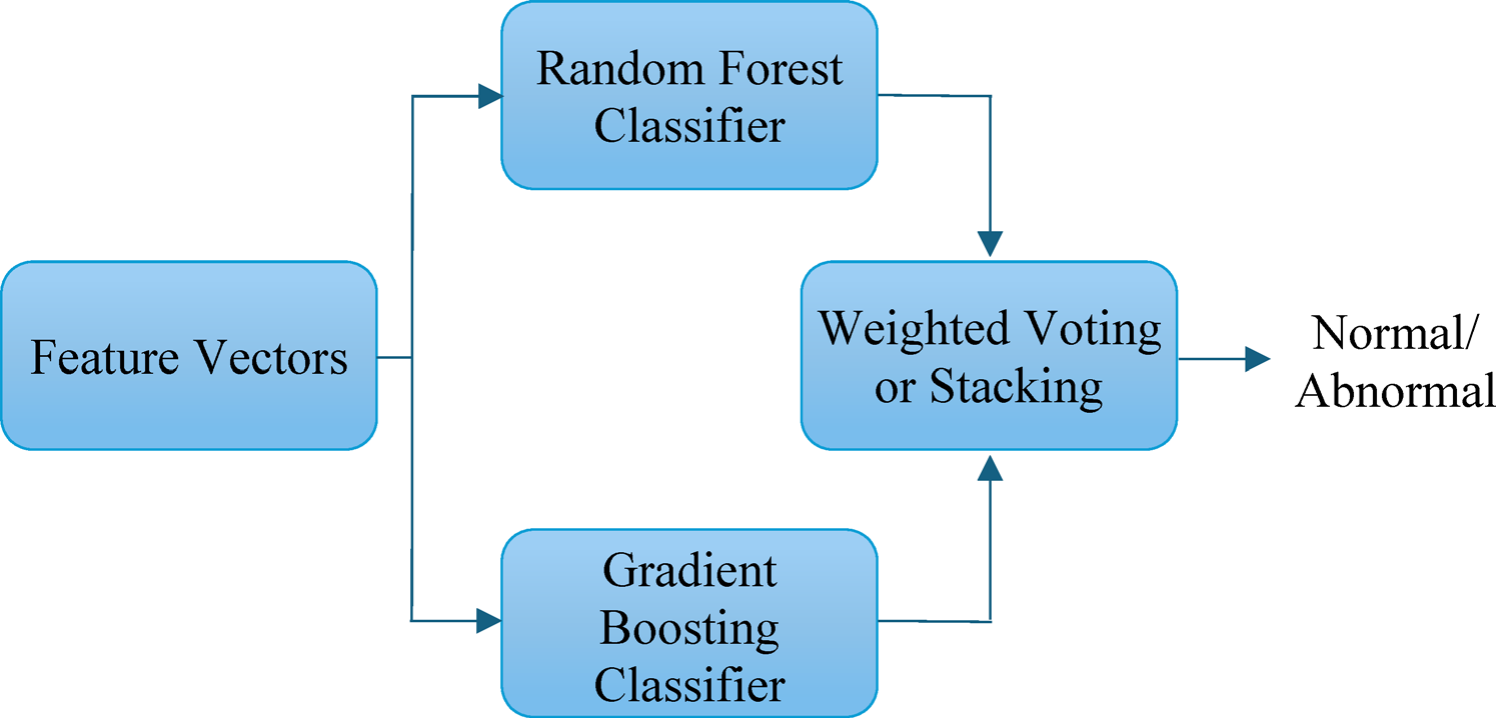
Structure of the hybrid ensemble classifier used for anomaly detection.

The overall structure is composed of the following components (see Fig. 3):

1. Feature Input Layer: The input to the ensemble consists of reduced-dimensionality feature vectors derived from spatial and temporal motion characteristics (e.g., magnitude, orientation, binary mask) extracted via optical flow from crowd regions detected by YOLOv7.
2. Base Classifiers:

- Random Forest (RF): Trained on the input features to provide robust predictions by averaging outputs from multiple decision trees. RF is effective in handling high-dimensional data and is resistant to overfitting.
- Gradient Boosting (GB): A sequence of weak learners (typically shallow trees) is trained iteratively, where each new learner corrects the errors of its predecessor. GB excels in capturing complex patterns and improving overall prediction accuracy.

3. Prediction Aggregation Mechanism: The predictions from RF and GB are aggregated using weighted voting, where each classifier’s output is assigned a weight based on its validation performance. Alternatively, a stacking strategy may be employed, where the predictions from RF and GB are used as meta-features and passed to a logistic regression layer for final classification.
4. Optimization Layer: The ensemble is optimized using the Adam optimizer, which adaptively adjusts learning rates for model parameters during training. This improves convergence speed and enhances generalization, especially on smaller datasets.
5. Output Layer: The final output is a binary classification, indicating whether the crowd behavior is normal or abnormal. This output is used to trigger alerts or visual annotations in a real-time surveillance system.

**Fig. 4:**
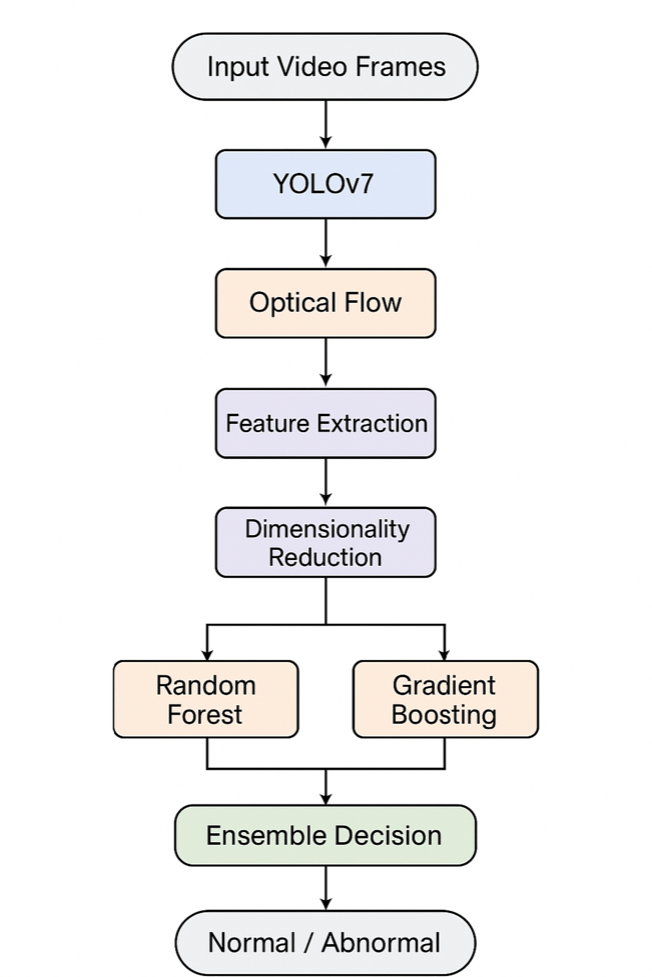
The architecture of the proposed hybrid ensemble anomaly detection framework.

Figure 4 presents the architecture of the proposed hybrid ensemble anomaly detection framework. The pipeline begins with video frame input, followed by crowd region detection using YOLOv7. Optical flow is then applied to capture motion dynamics, and a set of discriminative spatial–temporal features is extracted. To reduce computational complexity and eliminate redundancy, dimensionality reduction techniques are applied before classification. The processed features are subsequently passed to two base classifiers, Random Forest and Gradient Boosting, whose complementary strengths are leveraged through an ensemble decision mechanism. The final output is a binary classification of the observed behavior, indicating whether it is normal or abnormal. This layered architecture not only ensures robustness and interpretability but also enables the system to generalize effectively across both benchmark and real-world surveillance datasets.

### Proposed algorithm

**Fig. 5:**
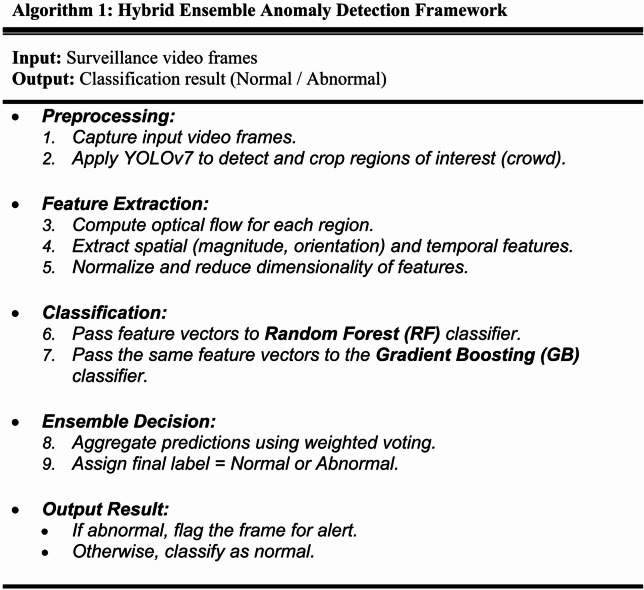
The pseudocode of the proposed framework algorithm.

To improve clarity and reproducibility of the proposed framework, we provide a step-by-step description of the complete workflow in Algorithm 1 (see Fig. 5). While the methodology has already been described in detail in earlier sections, presenting the framework in algorithmic form ensures a concise and unambiguous representation of the process.

As shown in Algorithm 1, the system begins with preprocessing and detection, where YOLOv7 identifies regions of interest in surveillance frames. Next, motion-based features are extracted using optical flow, capturing both spatial and temporal dynamics of the crowd. These features are then passed to two base classifiers—Random Forest and Gradient Boosting—which operate in parallel. Finally, an ensemble decision mechanism aggregates its outputs using weighted voting to produce the final classification of each frame as normal or abnormal.

### UMN dataset

**Fig. 6:**
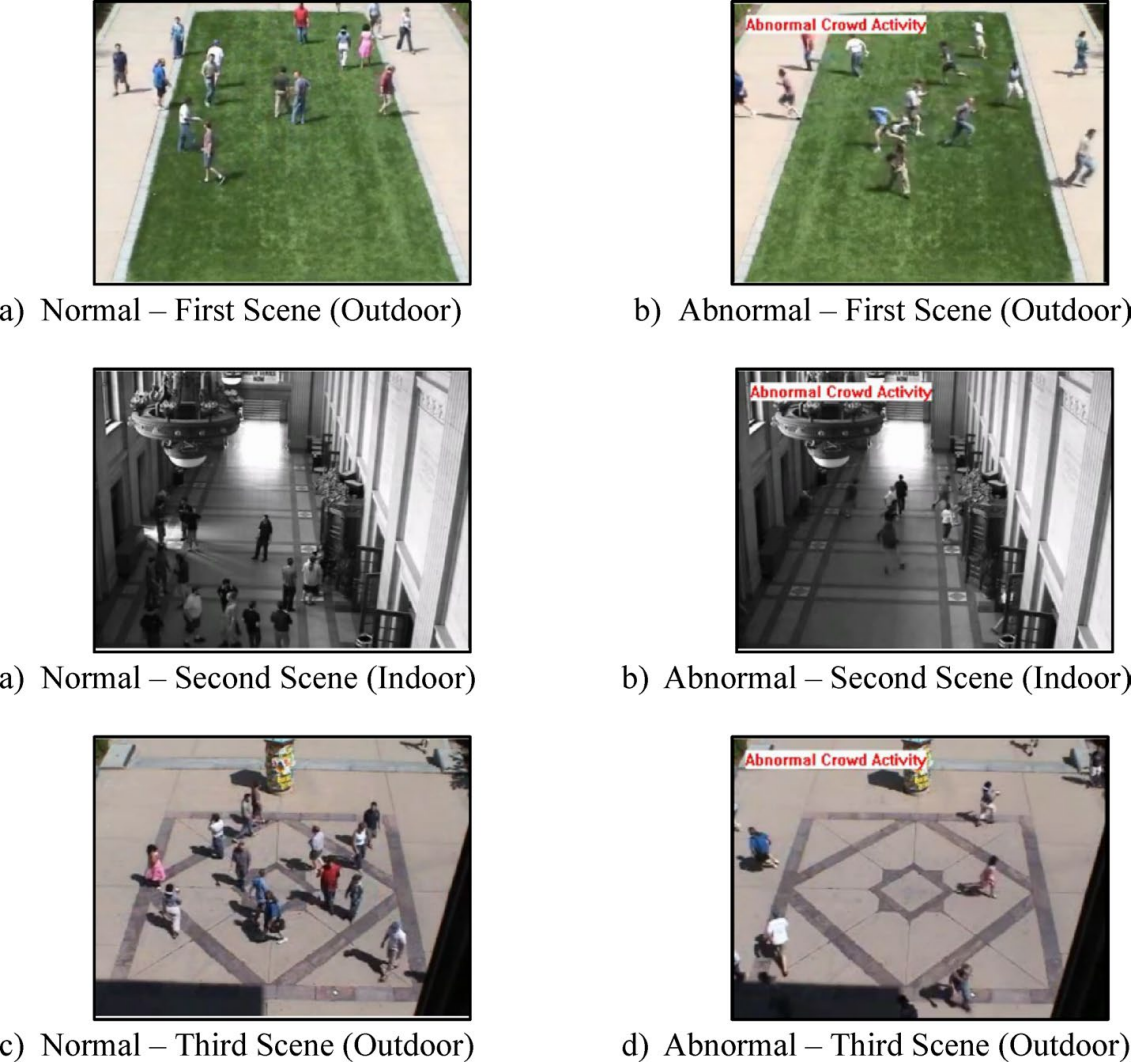
Sample frames of normal and abnormal behaviors of the UMN Dataset (three different scenes).

The UMN dataset^32^ is one of the most widely used datasets for research on crowd behavior analysis, anomaly detection, and related applications. It contains several video sequences captured in various public places, including pedestrian walkways and crowded road crossings. There are videos of 11 variations for each escape event in three indoor and outdoor scenarios. For example, walking is normal, but running is considered abnormal. The resolution of the frames in the UMN scenes is 320 × 240 pixels. Figure 6 shows sample frames for these scenarios. The left side displays normal behaviors in three different scenes, while the right side shows abnormal behaviors in three separate scenes. Each video begins with a brief clip of normal behavior and ends with sequences of abnormal behavior. These videos have been selected to encompass a wide range of normal and abnormal crowd behaviors that typically occur; therefore, the dataset is highly useful for researchers in the fields of computer vision and machine learning. Results from the UMN dataset have contributed to the growing research in crowd anomaly detection. Additionally, normal and abnormal behaviors were annotated in the dataset to enable the easy training and evaluation of supervised learning models.

### Custom supermarket surveillance dataset

#### Dataset description

**Fig. 7:**
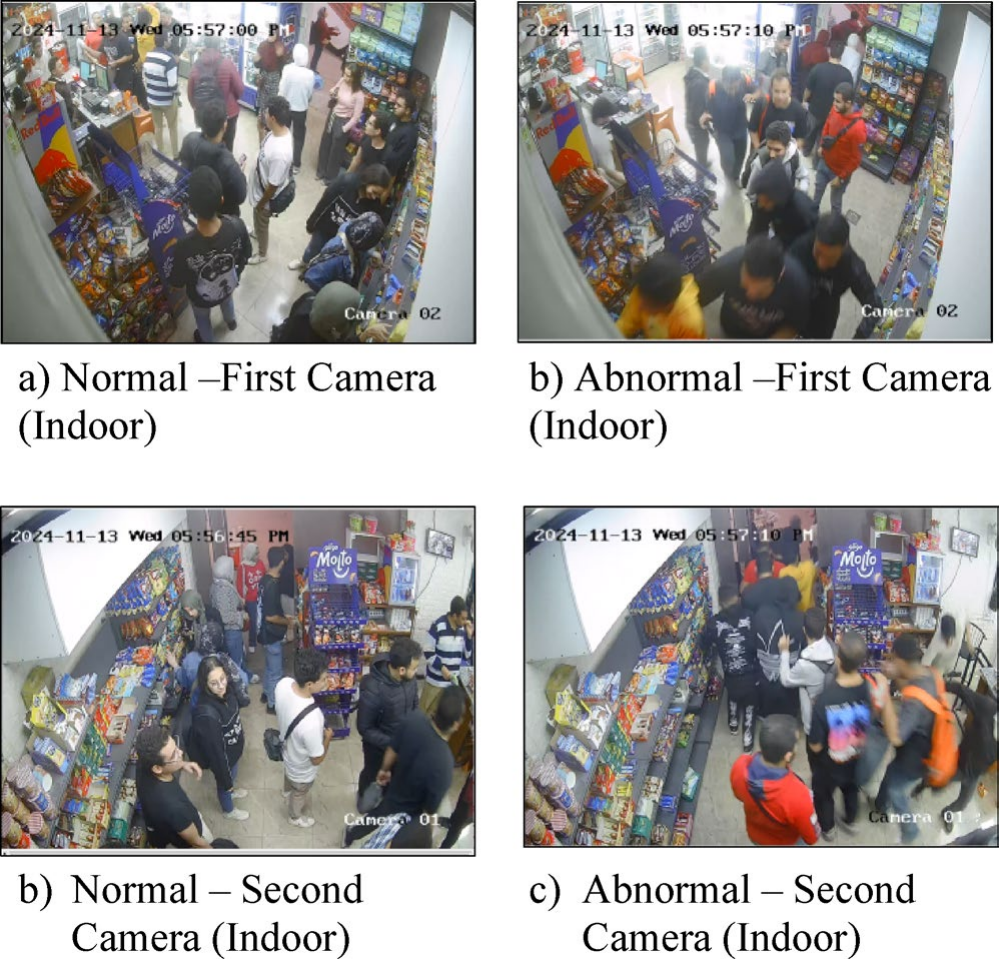
Sample frames of normal and abnormal behaviors of the custom supermarket surveillance dataset.

The custom dataset was developed to provide a real-world testing ground for the proposed hybrid anomaly detection system. It comprises surveillance footage from a supermarket environment, capturing both normal and abnormal activities in crowded scenes. Figure 7 shows sample frames for the dataset. The left side displays normal behaviors in two different cameras, while the right side shows abnormal behaviors. Key attributes of the dataset are:

- **Content**: The dataset comprises 250 video clips, featuring a balanced distribution of normal and anomalous activities.
  - *Normal activities*: Examples include individuals walking, browsing shelves, and waiting in line at checkouts.
  - *Anomalous activities*: Examples include running, loitering, theft attempts, and abrupt movements indicative of panic or unusual behavior.
- **Duration**: Each clip ranges from **10 to 30 s**, ensuring variability in activity duration and crowd density.
- **Annotation**: All videos are manually labeled with frame-level annotations to differentiate normal and abnormal events.
- **Diversity**: The dataset features scenes with varying lighting conditions, camera angles, and crowd densities, simulating real-world surveillance challenges.
- **Resolution**: Videos are recorded in **720p resolution**, ensuring sufficient detail for anomaly detection while maintaining computational efficiency.

#### Importance to the proposed method

To rigorously evaluate the effectiveness of the proposed hybrid anomaly detection method, a custom dataset was developed, tailored specifically to address the limitations of existing benchmark datasets. Unlike generic datasets that often lack the environmental variability and contextual nuance found in real-world surveillance, this dataset was designed to simulate practical scenarios, particularly those involving indoor, small-scale crowd behavior. Its design not only supports robust model validation but also provides a foundation for performance optimization and generalization across diverse use cases. The significance of this custom dataset is highlighted through the following key aspects:

1. **Real-world applicability**: Unlike benchmark datasets, the custom dataset reflects the complexity and noise of real-world environments, including variations in lighting, occlusions, and background activities. This ensures the model’s robustness and adaptability to practical surveillance scenarios.
2. **Small-scale crowd focus**: Many existing datasets are designed for large-scale crowd monitoring. This dataset addresses the unique challenges of small-scale crowd behavior, which is common in indoor environments such as supermarkets, where subtle anomalies may be more complex to detect.
3. **Fine-tuning and evaluation**: The dataset’s diversity enables fine-tuning of the hybrid model’s parameters, including the hyperparameters of Random Forests and Gradient Boosting, as well as the Adam optimizer. This ensures optimal performance in detecting both subtle and overt anomalies.
4. **Benchmarking new use cases**: By introducing this dataset, we establish a benchmark for supermarket surveillance, a domain that is underrepresented in anomaly detection research.
5. **Generalization validation**: High performance on this dataset demonstrates the system’s ability to generalize beyond traditional benchmarks, such as the UMN dataset, reinforcing its practicality for real-world deployment.

The supermarket dataset, characterized by higher resolution and a broader spectrum of anomaly types, offers a more challenging and realistic testing environment than the UMN dataset. By conducting experiments on both datasets, we highlight the generalizability and robustness of our proposed approach across diverse scenarios. Table 2 provides a clear comparative reference, helping researchers understand the unique features of each dataset and their implications for crowd anomaly detection performance.

**Table 2:** A comparison between the UMN benchmark dataset and the custom supermarket dataset.

Feature	Supermarket dataset	UMN dataset
Resolution	High resolution 720p (1280 × 720) (as stated in the dataset description)	Lower resolution (320*240) pixels
Environment	Supermarket Indoor	Public Walkways
Scene Complexity	More complex scenes with varying lighting, clutter, and occlusions (as mentioned in “Diversity”)	Less complex scenes with controlled lighting and fewer occlusions
Anomaly Types	Diverse anomalies, including shoplifting, loitering, and suspicious object handling (as mentioned in “Anomalous activities”)	Primarily focused on sudden running and escaping behavior.
Crowd Density	Variable crowd densities, ranging from sparse to moderately dense (as mentioned in “Duration”)	Mostly dense crowds
Camera Perspective	Surveillance camera perspective with varying angles and zoom levels (as mentioned in “Diversity”)	Fixed camera perspective with a wide view

#### Dataset availability

To promote reproducibility and facilitate further research, the dataset will be made publicly available, along with its annotations and usage guidelines, upon request. This will contribute to the development of anomaly detection methods tailored to indoor surveillance applications. This dataset not only complements the benchmark results but also underscores the practical contributions of the proposed method in addressing real-world surveillance challenges.

To develop a model capable of detecting anomalous behaviors in crowds, we constructed a dataset derived from surveillance footage captured in a supermarket environment. This dataset encompasses a range of crowd behaviors within a semi-controlled setting, recorded using two high-resolution cameras strategically placed to monitor movement patterns, social interactions, and potential anomalies. The supermarket offers a diverse and realistic backdrop, making it an ideal environment for training and evaluating crowd anomaly detection models. Each video sequence is meticulously annotated to distinguish between normal and abnormal behaviors, such as sudden running, loitering, or irregular gatherings. Annotators involved in the labeling process were trained to accurately identify and classify these behaviors, ensuring the reliability of the dataset. This annotated dataset serves as a foundational component for training and fine-tuning the proposed model, enabling it to detect and respond to unusual crowd activity with precision in real-world surveillance scenarios.

## Experimental results and discussion

### Implementation environment

The suggested hybrid ensemble learning was executed using a Python version 3.9 programming environment, including installing some packages like “OpenCV-python, OpenCV-python-headless, torch, torchvision, scikit-learn, NumPy, and seaborn”. The results were obtained on a personal computer (PC) equipped with an Intel Core i7-13620 H 13th Generation processor operating at 2.40 GHz. The machine had 16 GB of installed RAM (15.7 GB usable) and a 64-bit operating system on an x64-based processor architecture. Storage is supported by an SSD-based hard drive, which ensures fast read and write operations, making it suitable for data-intensive tasks. The System is also equipped with an NVIDIA GeForce RTX 4050 GPU. This provided high parallel processing capabilities, which were essential for accelerating machine learning model training for Large-scale data handling and high-resolution visualizations. The quantitative results demonstrated that the proposed hybrid ensemble learning approach was highly effective and outperformed contemporary methods.

### Hyperparameter configuration

To ensure optimal performance and reproducibility, the proposed hybrid anomaly detection system was configured with carefully selected hyperparameters across all components, as shown in Table 3. The YOLOv7 object detection model was set with an input resolution of 640 × 640 pixels, a confidence threshold of 0.5, and a non-maximum suppression (NMS) IoU threshold of 0.45. Pre-trained weights were fine-tuned on both the UMN and the custom supermarket datasets. For the ensemble classification stage, the Random Forest classifier was configured with 100 decision trees and a maximum depth of 10, ensuring robustness without overfitting. The Gradient Boosting classifier was set with a learning rate of 0.1 and 100 boosting stages, allowing for the gradual refinement of predictions. Both classifiers were optimized using the Adam optimizer with default hyperparameters: a learning rate of 0.001, β₁ = 0.9, β₂ = 0.999, and ε = 1e-08. During training, a batch size of 32 was used, and the dataset was split into 70% training and 30% testing, which yielded the highest performance. Additional evaluations were conducted using 65/35 and 80/20 splits to assess stability. These settings were selected based on iterative experimentation and prior literature, striking a balance between computational efficiency and predictive accuracy.

**Table 3:** Overall system hyperparameters.

System component	Parameters
YOLOv7	• Input resolution: 640 × 640
	• Confidence threshold: 0.5
	• IoU threshold for NMS: 0.45
	• Pre-trained weights: YOLOv7 official weights fine-tuned on our datasets
Training Parameters for RF and GB	• Number of trees in Random Forest: 100
	• Maximum tree depth: 10
	• Learning rate for Gradient Boosting: 0.1
	• Number of boosting stages: 100
Optimizer Settings (Adam)	• Learning rate: 0.001
	• β1 = 0.9, β2 = 0.999 (default values)
	• ε = 1e-08
Training Setup	• Batch size: 32
	• Train/test splits: 70/30 (with experiments also on 65/35 and 80/20)
	• Number of training epochs: 100

### Evaluation metrics

To evaluate the performance of the proposed method, assessments are conducted across both spatial and temporal domains. In the spatial domain, key performance metrics used in the literature review include accuracy, precision, recall, and F1 score. These metrics are calculated based on the standard classification outcomes: True Positives (TP), True Negatives (TN), False Positives (FP), and False Negatives (FN), as defined below^33^:

4 $$\:accuracy=\:\frac{TP+TN}{TP+TN+FP+FN}$$

5 $$\:Precision=\:\frac{TP}{TP+FP}$$

6 $$\:Recall=\:\frac{TP}{TP+FN}$$

7 $$\:F1score=\:\frac{2\text{*}Precision\text{*}Recall}{Precision+Recall}$$

### Performance on UMN dataset

**Fig. 8:**
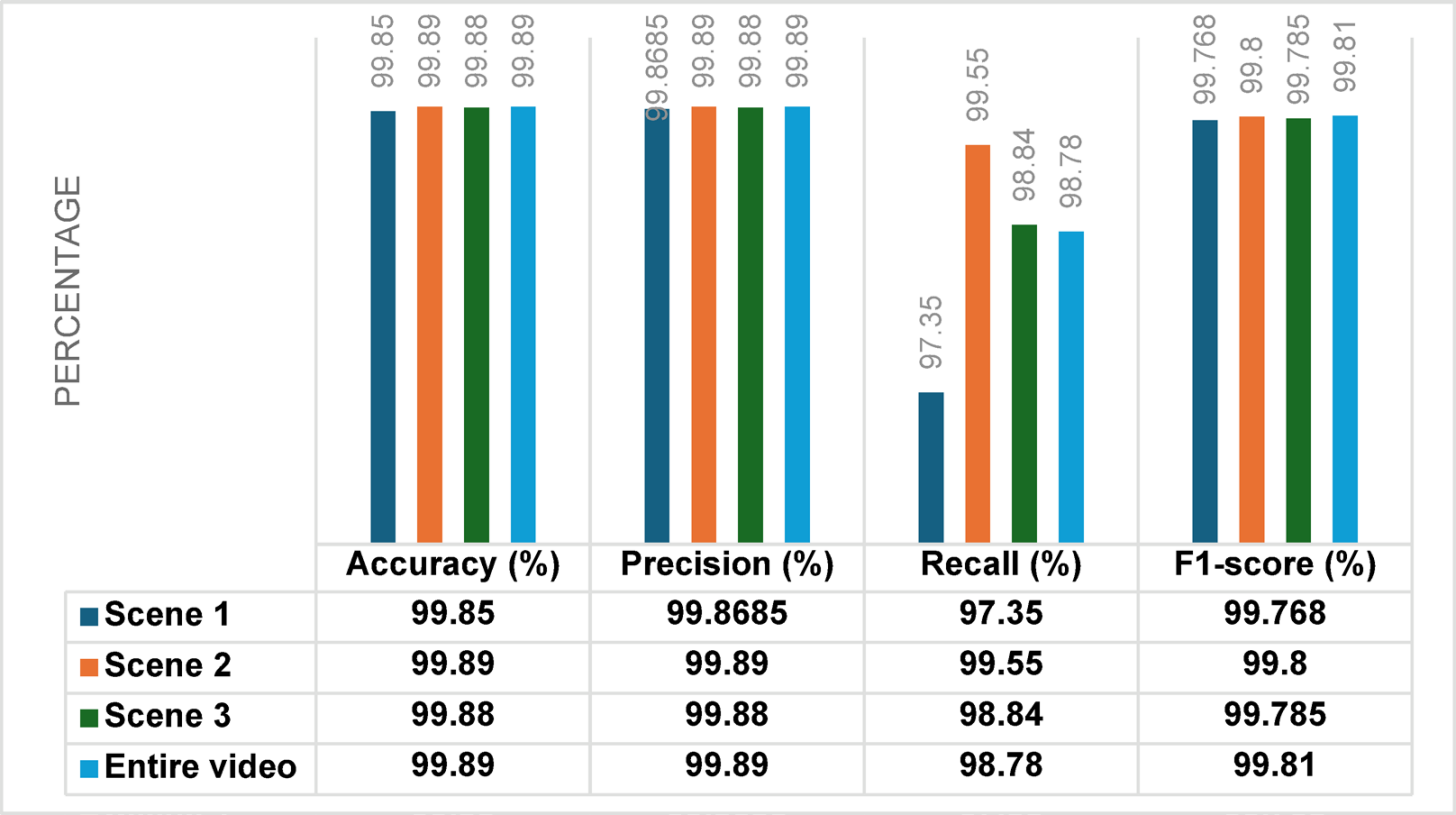
Comparative evaluation of the Proposed Method and Contemporary Approaches on the UMN Dataset.

**Fig. 9:**
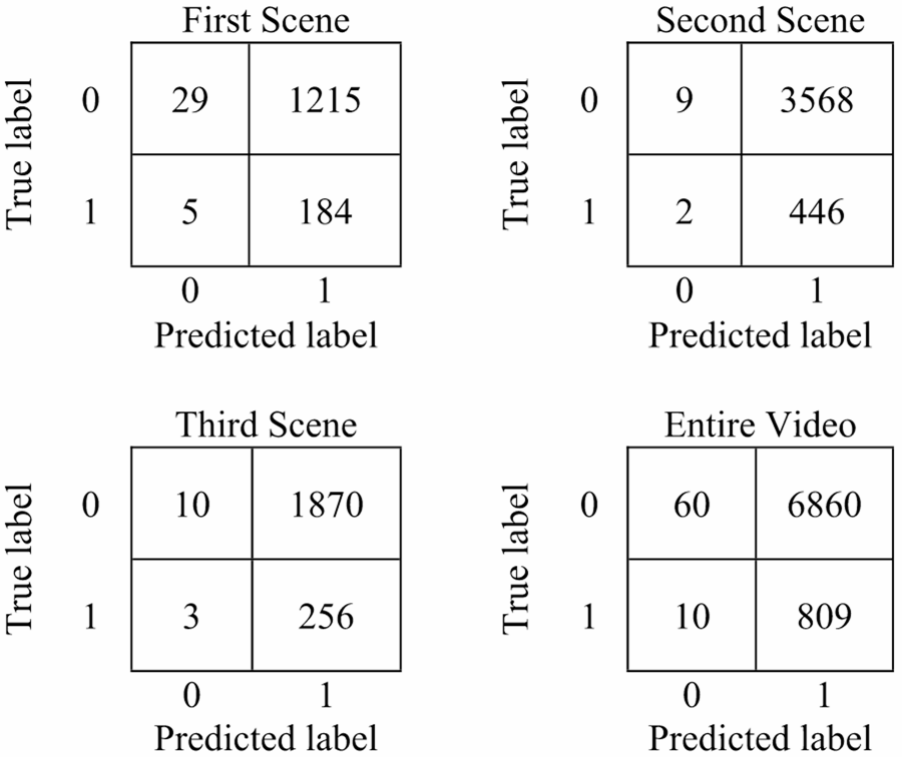
Confusion matrix for the UMN dataset.

The results of frame-level anomaly detection in the spatial domain using small-scale crowd datasets demonstrate the effectiveness of the proposed hybrid method. To ensure a robust evaluation, we systematically investigated various training-testing splits, including 80% training and 20% testing, 65% training and 35% testing, and 70% training and 30% testing. Our experiments revealed that the 70% training and 30% testing split yielded the best accuracy, showing an improvement of approximately 2% compared to other configurations. Utilizing this optimal partitioning, when evaluated on the UMN dataset, the model achieved exceptionally high accuracies of 99.85%, 99.89%, and 99.88% across scenes 1, 2, and 3, respectively. These results are summarized in Table 4. Figure 8 presents the results in a bar chart, highlighting the robustness and consistency of the hybrid classifier across various crowd scenarios. The corresponding confusion matrix for the UMN dataset, illustrated in Fig. 9, further confirms the model’s strong discriminative capability, showing minimal false positives and false negatives. The outstanding performance can be attributed to the complementary strengths of the integrated RFs and GB classifiers, where RFs contribute to noise resistance and feature handling. GB enhances fine-grained decision boundaries. Moreover, the careful feature extraction and preprocessing stages, along with fine-tuned hyperparameters, enable the model to generalize effectively across different crowd dynamics and environmental conditions.

**Table 4:** The results of a hybrid method on the UMN dataset.

Dataset/scene	# frame size	Accuracy (%)	Precision (%)	Recall (%)	F1-score (%)
Scene 1	1433	99.85	99.8685	97.35	99.768
Scene 2	4025	99.89	99.89	99.55	99.8
Scene 3	2139	99.88	99.88	98.84	99.785
Overall	7739	99.89	99.89	98.78	99.81

### Comparison with state-of-the-art methods

To ensure a fair and unbiased comparison, all baseline methods and the proposed hybrid framework were trained and evaluated under identical conditions. The same datasets, preprocessing steps, train-test splits, and evaluation metrics were applied uniformly across all experiments. Furthermore, all experiments were executed on the same hardware and software environment, and hyperparameters for baseline methods were either adopted from their original studies or tuned using the same validation protocol. This consistency guarantees that the observed performance differences reflect the inherent capabilities of the methods rather than variations in the experimental setu.

**Fig. 10:**
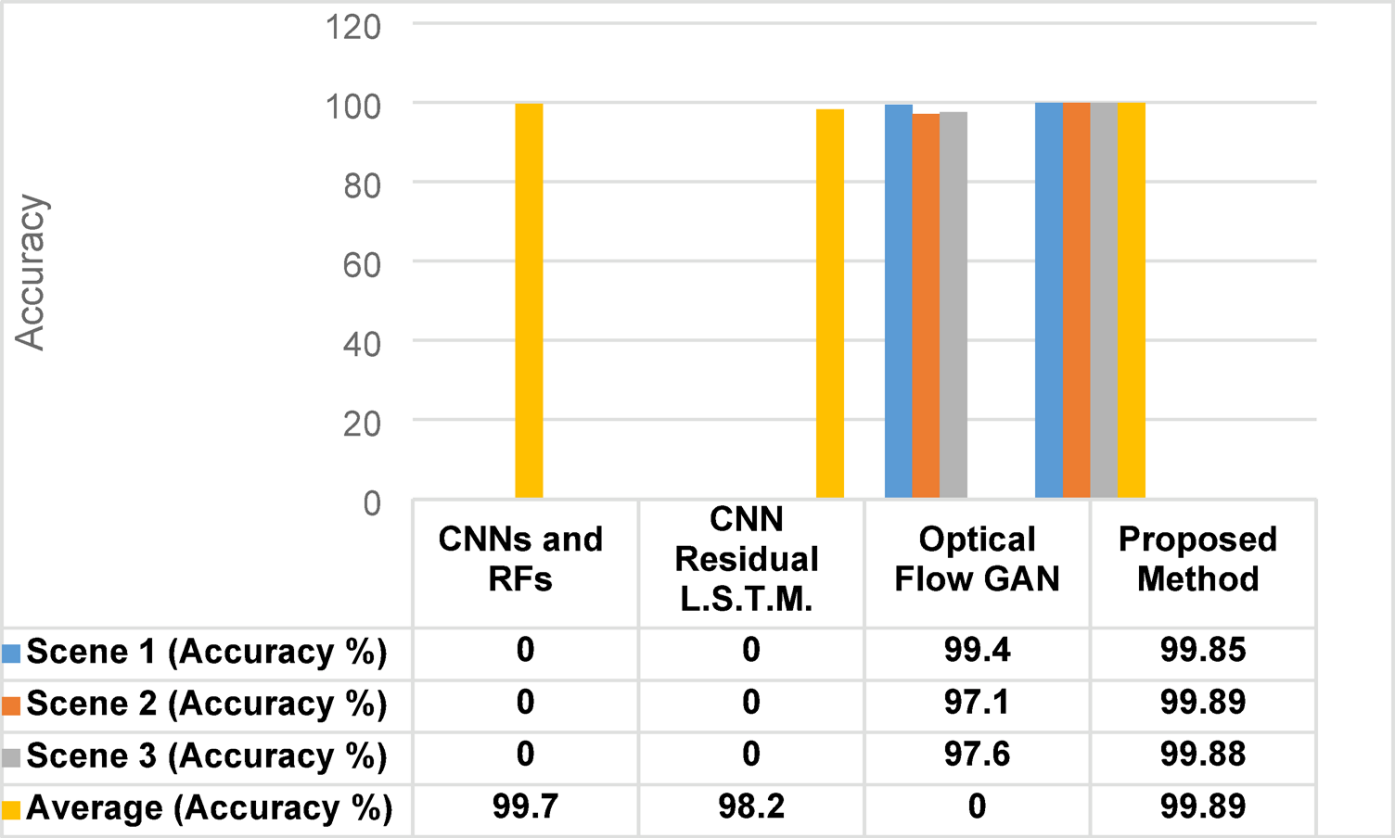
Accuracy comparison of the proposed method with state-of-the-art approaches on the UMN dataset. All methods were evaluated under identical experimental settings (datasets, train/test splits, preprocessing, and metrics) using the same hardware/software environment, ensuring that performance differences are attributable to the inherent capabilities of the methods.

To provide a relative evaluation of the proposed method, a comparison is conducted with other recent state-of-the-art CAD methods, namely CNNs combined with RFs^17^, CNN Residual LSTM^34^, and Optical Flow GAN^35^. These works were selected as they represent strong baselines with competitive performance on the UMN dataset and are widely cited in the field. As shown in Table 5, the proposed hybrid method significantly outperforms existing approaches on the UMN dataset, achieving scene-wise accuracies of 99.85%, 99.89%, and 99.88%, with an overall accuracy of 99.89% across the entire video. Figure 10 shows the overall accuracy comparison of different methods. These results surpass those of previous state-of-the-art methods, such as CNNs combined with RFs (99.77%), CNN Residual LSTM (98.2%), and Optical Flow GAN, which reported scene-wise accuracies of 99.4%, 97.1%, and 97.6%. The superior performance of the proposed method can be attributed to the integration of RFs and GB classifiers, which complement each other by combining robustness to noise with high predictive precision. Furthermore, the use of effective feature extraction techniques, along with fine-tuned hyperparameters and optimized learning through the Adam optimizer, enables the model to capture both subtle and abrupt behavioral changes in crowd dynamics. These enhancements enable the system to generalize more effectively across varying conditions and scene complexities, thereby increasing its reliability for real-world surveillance applications.

**Table 5:** Scene-wise and average accuracy (%) comparison between the proposed method and other state-of-the-art approaches on the UMN dataset. All methods were evaluated under identical experimental settings (datasets, splits, preprocessing, and metrics) on the same hardware/software platform, ensuring that performance differences reflect methodological capabilities rather than setup variations.

Ref.	Method	Scene 1 (Accuracy %)	Scene 2 (Accuracy %)	Scene 3 (Accuracy %)	Average (Accuracy %)
^17^	CNNs and RFs	-	-	-	99.7
^34^	CNN Residual L.S.T.M.	-	-	-	98.2
^35^	Optical Flow GAN	99.4	97.1	97.6	-
--	Proposed Method	99.85	99.89	99.88	99.89

We have designed a hybrid method for detecting abnormal behavior in crowded scenes using small-scale crowd videos by combining random forests and gradient-boosting classifiers. The results of experiments on the UMN dataset demonstrated that the proposed hybrid method is effective in identifying anomalies within crowded scenes. The better accuracy of the proposed method compared to both state-of-the-art techniques and even the RF classifier justifies the potential of this approach. The key integration of the RFs and GB classifiers likely contributed to improving the performance of the classifier, as the Random Forest provides a great starting point for the model, inherently handling complex patterns while reducing overfitting. It also involves a Boosting technique: a self-improving model that learns sequentially from misclassified instances to enhance its ability for better anomaly detection. Currently, the model can only classify the crowd scenes as either normal or abnormal. However, to increase its applicability in practical scenarios, future research should be able to identify the type of abnormal behavior, where it occurs in the scene, and in what temporal context it happens. Such fine details would enable more accurate interventions and highly specific responses to potential threats or incidents.

### Performance on custom supermarket dataset

To comprehensively assess the effectiveness of the proposed method, further evaluation was conducted using our newly developed real-world supermarket surveillance dataset. While the UMN dataset primarily captures specific anomaly types in controlled outdoor environments with consistent resolution, the custom supermarket dataset introduces a more complex and dynamic setting characterized by indoor scenes, varying crowd densities, and diverse environmental conditions. This inclusion allows for a more rigorous assessment of the model’s generalizability and robustness. When applied to the supermarket dataset, the proposed hybrid method demonstrated its effectiveness by achieving an overall accuracy of 92.6%, with a precision of 91.8% and a recall of 93.3% in anomaly detection. The corresponding confusion and performance matrices are presented in Tables 6 and 7, which demonstrate the model’s ability to reliably distinguish between normal and abnormal behaviors under real-world surveillance conditions. These results affirm the adaptability of the proposed approach to practical scenarios beyond benchmark datasets.

Notably, the system performed exceptionally well in detecting overt anomalies, such as running or theft attempts, achieving nearly perfect classification. However, subtle anomalies, such as loitering, posed slightly greater challenges, contributing to a minor drop in precision. This highlights the need for further refinement in identifying less pronounced behavioral patterns.

**Table 6:** The confusion matrix of running the proposed method on the custom supermarket surveillance dataset.

	Actual positive	Positive negative
Classified Positive	TP 260,000	FP 23,224
Classified Negative	FN 18,670	TN 17,800

**Table 7:** The performance matrix of running the proposed method on the custom supermarket surveillance dataset.

Metric	Value (%)
Precision	91.8
Recall	93.3
Accuracy	92.6

It is important to emphasize that the differences observed between the UMN benchmark dataset and the custom supermarket dataset stem from their intrinsic characteristics rather than variations in experimental setu The UMN dataset represents a controlled environment with limited anomaly types (primarily running/escaping), low resolution (320 × 240), and fewer occlusions, which naturally leads to higher performance. In contrast, our supermarket dataset was deliberately designed to capture real-world complexities, including higher resolution (720p), varying lighting, diverse crowd densities, and subtle anomalies such as loitering or shoplifting. These conditions introduce additional challenges and explain the slightly lower accuracy (92.6%). Nevertheless, the high recall achieved demonstrates the model’s robustness in minimizing false negatives, which is critical for real-world surveillance. By evaluating the proposed method under both controlled and real-world conditions with identical preprocessing, model configuration, and evaluation metrics, we validate its adaptability and generalization capability.

Overall, the results validate the adaptability and generalization capability of the proposed system, underscoring its potential for deployment in practical scenarios such as supermarket monitoring. The custom dataset thus provides valuable insights into the model’s strengths and areas for improvement, contributing to future enhancements in anomaly detection.

### Time complexity analysis

To assess the efficiency of the proposed system, we conducted a time-complexity analysis across both the UMN and the custom supermarket surveillance datasets. The processing pipeline consists of three main stages: YOLOv7-based crowd detection, optical flow-based feature extraction, and classification using a hybrid ensemble learning approach (Random Forest and Gradient Boosting). On average, the system processes each frame in 42 ms for the UMN dataset, translating to approximately 24 frames per second, which meets the criteria for near real-time inference. For the supermarket dataset, which contains higher-resolution frames (720p) and more complex scenes, the average processing time was 64 ms per frame, resulting in approximately 15.6 frames per second. These measurements were obtained using a system equipped with an Intel Core i7-13620 H CPU and an NVIDIA GeForce RTX 4050 GPU. From an algorithmic standpoint, YOLOv7 contributes O(n) complexity with respect to the number of objects per frame, while the feature extraction and ensemble classification components operate with O(d⋅log d) complexity, where d is the dimensionality of extracted features. These results demonstrate the framework’s suitability for deployment in real-world surveillance environments, particularly those that require fast and reliable anomaly detection. Table 8 summarizes the processing time and frame rate for each dataset.

**Table 8:** Time-complexity evaluation on benchmark and custom datasets.

Dataset	Resolution	Avg. frame processing time (ms/frame)	Frame rate (FPS)	Total runtime (for full dataset)
UMN Dataset	320 × 240	42 ms	~ 24 FPS	~ 5.4 min for 7,739 frames
Custom Supermarket Dataset	1280 × 720	64 ms	~ 15.6 FPS	~ 4.3 min for 4,140 frames

As observed, the UMN dataset benefits from lower resolution and simpler scenes, enabling near real-time processing at ~ 24 FPS. In contrast, the custom supermarket dataset, with higher resolution and more complex scenes, results in a slightly slower frame rate (~ 15.6 FPS). This demonstrates the scalability of the proposed framework across environments with varying levels of complexity.

While the proposed system demonstrates strong performance in detecting abnormal behaviors at the frame level, it does not explicitly localize where within a frame the anomaly occurs. In our current design, YOLOv7 is used to identify crowd regions and individuals, but the ensemble classifiers operate at the frame level to provide a binary anomaly decision. This was an intentional design choice to prioritize robust and generalizable anomaly detection across diverse datasets. Nonetheless, we acknowledge that precise spatial localization of anomalies would significantly enhance the interpretability and practical utility of the framework. As part of our future work, we plan to integrate region-proposal or spatio-temporal attention mechanisms that can associate detected anomalies with specific bounding boxes or sub-regions in the scene, thereby enabling more fine-grained anomaly localization.

## Conclusion and future work

This paper presented a hybrid anomaly detection framework designed to address persistent limitations in crowd anomaly detection, particularly in real-world, small-scale, and indoor surveillance environments. While prior work has leveraged deep models or single-classifier pipelines, our approach uniquely combines YOLOv7 for efficient region-of-interest detection with a dual-path ensemble classifier consisting of Random Forest and Gradient Boosting models. This structure strikes a balance between accuracy, interpretability, and computational efficiency—three challenges that are often unmet in the current literature.

Through extensive evaluation on both the UMN benchmark dataset and a custom supermarket surveillance dataset, our model demonstrated state-of-the-art performance, achieving up to 99.89% accuracy in controlled settings and 92.6% accuracy in complex real-world scenarios. These results affirm the system’s ability to generalize across environments, crowd behaviors, and scene complexities.

The novelty of this work lies in its hybrid integration of interpretable and powerful classifiers, its optimization for small and noisy datasets, and its practical deployment potential in live surveillance contexts. Future research will focus on extending the model to perform fine-grained anomaly classification and localization, enabling actionable insights for security operators. We also aim to adapt the system for real-time edge deployment, addressing hardware constraints while maintaining optimal detection performance.

## Data Availability

The dataset analyzed during the current study is available at [https://mha.cs.umn.edu/proj_events.shtml#crowd](https:/mha.cs.umn.edu/proj_events.shtml). Additionally, the Real dataset, “Custom Supermarket Surveillance,” is available from the corresponding author upon reasonable request.If you want to access the private real dataset, please contact the corresponding author through email: [Dmabrouk@ecu.edu.eg](mailto: Dmabrouk@ecu.edu.eg).
